# Preparation and Mechanical Properties of Microcapsule-Based Self-Healing Cementitious Composites

**DOI:** 10.3390/ma14174866

**Published:** 2021-08-27

**Authors:** Shiping Jiang, Zhiyang Lin, Can Tang, Wenfeng Hao

**Affiliations:** 1 Faculty of Civil Engineering and Mechanics, Jiangsu University, Zhenjiang 212013, China; 1000004644@ujs.edu.cn (S.J.); linzhiyang@jsslsj.com (Z.L.); hwf2016@ujs.edu.cn (W.H.); 2 National Center for International Research on Structural Health Management of Critical Components, Jiangsu University, Zhenjiang 212013, China; 3 Jiangsu Surveying and Design Institute of Water Resources Co., Ltd., Yangzhou 225127, China

**Keywords:** self-healing cementitious composites, mechanical property, strength healing performance, age of concrete, microcapsule content

## Abstract

Self-healing concrete designs can protect against deterioration and improve durability. However, there is no unified conclusion regarding the effective preparation and mechanical properties of self-healing concrete. In this paper, microcapsules are used in cement-based materials, the reasonable dosage of microcapsules is determined, and the self-healing performance of the microcapsule self-healing system under different curing agents is explored. The microcapsules and curing agent are shown to enhance the flexural and compressive strength of mortar specimens at relatively low contents. The optimal microcapsule content in terms of compressive strength is 1–3%. When the content of the microcapsule reaches 7%, the strength of the specimen decreases by approximately 30%. Sodium fluorosilicate is better-suited to the microcapsule self-healing cement-based system than the other two fluorosilicates, potassium fluorosilicate and magnesium, which have similarly poor healing performance as curing agents. Healing time also appears to significantly influence the microcapsule self-healing system; mortar specimens that healed for 28 days are significantly higher than those that healed for 7 days. This work may provide a valuable reference for the design and preparation of self-healing cementitious composite structures.

## 1. Introduction

Self-healing concrete is a workable approach to preventing deterioration and improving durability [1,2,3,4]. It is difficult to secure sufficient manpower and material resources for damage detection and timely repair in the use of concrete components. Self-healing concrete materials with bionic characteristics have been developed as a potential solution to this problem. Among them, microencapsulated self-healing composites have garnered a great deal of research attention in regards to cement-based materials [5,6,7,8].

Researchers have established many different self-healing concrete methods and mechanisms. Existing self-healing concrete formulations can be roughly split into two categories: autonomous and autogenous [9,10]. Autonomous healing concrete designs mainly emphasize the self-healing potential of the concrete itself, that is, further hydration of cement-based materials such as carbonation and pozzolanic reaction [11,12]. However, the healing ability provided by the matrix itself is usually insufficient to compensate for further expansion and extension of cracks in the concrete.

Autogenous healing mainly refers to artificial designs, where additional components are supplemented with the specific purpose of healing. These designs include non-encapsulated crack self-healing, encapsulated self-healing, and microbial self-healing. Microencapsulated self-healing concrete has passive encapsulation. The repairing agent is mainly composed of resin or chemical crystal. With the destruction of the concrete, the core material of the crack flows out, and the crack is filled and healed [13]. Various factors, including the core, wall, particle size, and curing agent, affect the healing properties of the material [14]. Microencapsulated self-healing concrete has gradually progressed from the laboratory to the engineering field, for example, in a tunnel project in Shenzhen [15]. Engineers in the UK conducted the first large-scale field test of self-healing concrete over four types of technology including microencapsulated self-healing concrete [16].

Generally speaking, most capsule methods are either chemical-based or biological-based. The self-healing behavior of the capsule method must be secured by a chemical reaction between the core material and the outside environment [17,18,19]. Healing depends on the process of packaging container damage in this case, so it is classified separately; it formally imitates the process of cell blood vessel rupture and releases healing substances after the organism is injured.

Capsule methods can be based on liquid tubes and microcapsules according to the type of container they utilize. In the liquid tube method, the main restorants are organic polymer materials. Other studies on microcapsule methods have used inorganic materials as restorants [10,20,21,22]. Dry et al. [23,24] first proposed that polypropylene pipes with adhesive should be put into concrete. When cracks occur in the concrete, the healing agent is released by heating as the curing agent heals the material so as to improve its permeability and strength. Glass fiber pipes with acetal polymer solution can also be added into the concrete to enhance its performance. This particular self-healing method can significantly improve the flexural strength and ductility of concrete.

Beglarigale et al. [25] established an orientation function describing the distribution of capsules in concrete, determined the failure stress of repaired capsules, and obtained the optimal capsule volume, volume ratio, wall thickness, and other parameters through a combination of finite element analysis and experiments. They adopted different adhesives and observed the balance states in three-point bending tests based on differential elements to determine the repair ability of a liquid pipe self-healing concrete beam. Zhan et al. [26] embedded a hollow fiber tube containing a repair agent into a cement mortar matrix and tested repaired specimens with an Instron testing machine and acoustic emission instrument. They observed significant improvement in the fracture performance of mortar.

In this study, self-healing microcapsules with different components were prepared by extrusion and round spray drying. Three kinds of fluorosilicates that may react with sodium silicate were introduced into cement mortar as curing agents. The basic mechanical properties of three kinds of repair agents with different microcapsule contents were compared and the strength recovery performance of mortar specimens under complete failure conditions was tested. A reasonable microcapsule content range and the optimal type of healing agent for the cement mortar matrix were determined accordingly.

## 2. Materials and Methods

### 2.1. Microcapsule Preparation

The core and wall of microcapsules are their most important parts. The core wall ratio is an important factor in preparing self-healing microcapsules with high efficiency by physical spraying and should be improved to the greatest extent possible within an appropriate range. This ratio also ensures that the broken microcapsules have an adequate healing effect in the cracked concrete and that microcapsules without cracks are not destroyed. Both must have a certain level of chemical stability and not react with adjacent media [27,28,29].

The capsule wall material must satisfy strict sealing requirements to prevent the core material from flowing outward and reacting in an unbroken state. It also must have a strong film-forming ability, which is mainly reflected in the preparation process. The wall material should be well-sprayed and attached to the surface of the core material in the microcapsule preparation process. The wall also must have sufficient strength to prevent the core material from flowing out in advance of concrete preparation, but not excessive strength so that the microcapsule is not damaged when the concrete cracks. The bond strength between the capsule wall and the concrete also must exceed that between the capsule wall and the core material to avoid cracks in the excessive area between them and to maintain consistency with the mechanism of the microcapsule self-healing concrete [30,31,32].

Sodium silicate is compatible enough with cement-based materials to react with them in the presence of water. It is also a common mortar waterproofing agent and has many applications in corrosion-resistant engineering and septic anti-seepage components. However, the direct addition of unmodified sodium silicate into concrete has some adverse effects [33,34]. Large amounts of sodium silicate produce abundant colloids and non-reactive free water in the concrete, which creates micro-cracks due to volume change after curing and drying shrinkage and drives down the concrete’s impermeability. In addition, as a type of sodium salt, sodium silicate may be harmful to the concrete aggregates (typically mortar materials).

The core wet material is comprised of an emulsifier and a thickener, both of which are typically polymers that are not chemically compatible with cement-based materials and thus must be carefully controlled. Sodium silicate and expansive Portland cement have a certain consistency and viscosity, which minimizes the necessity for organic materials. Through a series of pre-experiments, the minimum proportion that satisfies spheronization was obtained. At this ratio, most of the core materials are still capable of self-healing.

The wall material should be colloid or dextrin to satisfy the requirements of microencapsulation technology; however, these materials have poor hydrophilicity. The capsule wall performance also must be taken into account. Ethyl cellulose was used in this study as the capsule wall material. Ethyl cellulose is a common water-retaining agent and stabilizer that is often used as a coating material to make sustained-release drug capsules. It has strong adhesion, film-forming and filling functions, and relatively stable chemical properties; it is insoluble in water in alkaline environments, which allows it to effectively seal capsule walls [35]. It is stronger than other colloids, which can ensure that microcapsules are not damaged in the process of concrete production.

A WJ-3 extrusion spheronizer (Changzhou Panfeng Drying equipment Co., Ltd., Changzhou, China) was used to prepare sample microcapsules. Wet material was prepared, and strips were extruded, followed by rolling balls, spray coating, and hot air drying. The first three steps are mainly focused on pellets, then spray coating combines the core and the wall. The coating solution must be allocated properly prior to this process. Due to the relatively high viscosity of the ethyl cellulose solution used in the capsule wall, the dissolving agent has volatile components and a thermosetting phenomenon, so hot air drying is necessary to reduce the mutual adhesion. The preparation of wet material is the most important part of this process, as it directly determines the composition of repair material in the core material.

(1) Wet material preparation: The specific gravity of the water in wet material is 30%. The powder is comprised of Portland cement, ground sodium silicate, microcrystalline cellulose, and methyl cellulose mixed evenly in proportion with a cement mortar mixer. Distilled water and TWEEN 80 required by the proportion of humidity are evenly vibrated by an ultrasonic oscillator and rapidly mixed to ensure the proper humidity.
(2) Extrusion and spheronization: The prepared wet material is slowly poured into a running extrusion system. After extrusion, it falls directly into a working spheronizer. The rotational speed of the spheronizer is controlled between 500–1000 revolutions. The interruption system and centrifugal effect of the spheronizer gradually interrupt the extruded cylindrical strip, then spheronizes and spheroidizes it. In the extrusion process, the diameter of the small holes in the sieve plate is set to about 1 mm to control the bottom diameter of the extruded strip product.
(3) Coating: The ethyl cellulose powder is dissolved in the mixed solution of xylene and ethanol in the proportions necessary to form a coating solution. The material is sprayed into a roller with a spray gun, then the rotation speed of the spheronizer is reduced until it is stopped once most of the microcapsules have a significant luster and make a crisp sound upon impact. When the package is finished, the material is completely dried at a temperature of 30 °C. The microcapsule prepared by this method has sufficient encapsulation and strength to satisfy the working mechanism of microcapsule self-healing concrete.
(4) Screening process: Microcapsules with certain defects or oversize particles are screened out to retain only those 1.25–1.5 mm in size.

The main wall materials and core materials are ethyl cellulose M70, absolute ethyl alcohol, xylene, and sodium silicate hydrate. The emulsifier and dissolving agent in the capsule are hydroxypropyl methyl cellulose, microcrystalline cellulose, TWEEN 80 solution, and distilled water.

**Figure 1:**
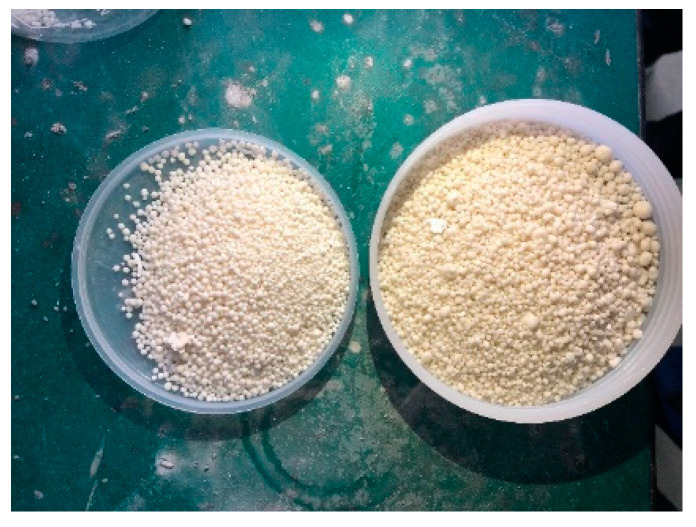
Microcapsules.

Portland cement and sodium silicate, which can react with calcium hydroxide, were embedded simultaneously in this experiment. Self-healing microcapsules with sodium silicate and expansive Portland cement as core materials were also fabricated for the sake of comparison. As mentioned above, preliminary experiments were run to determine the proper core material humidity, emulsifier quantity, and thickener quantity. The proportions of adhesive and expansion components in the self-healing component were adjusted accordingly. The healing component sodium silicate and expansive Portland cement were prepared at ratios of 3:7, 1:1, and 7:3 (that is, with sodium silicate proportions of 30%, 50%, and 70%, respectively). The microcapsules are shown in Figure 1.

### 2.2. Composite Preparation

(1) Cement: The physical properties of Helin ordinary Portland cement P O 42.5R are shown in Table 1 and Table 2.
(2) Fine aggregate: Chinese ISO standard sand was used with a fineness modulus of 2.4.
(3) Curing agent: Analytical pure sodium fluorosilicate, potassium fluorosilicate, and magnesium fluorosilicate (Sinopharm Chemical Reagent Co., Ltd., Shanghai, China) were used. The preparation time of these three different curing agents is denoted as FNA, FK, and FMG, respectively.
(4) Water: Ordinary tap water from Zhenjiang Jiangsu was used. The pH of the water is about 6.5.

The microencapsulated self-healing cement mortar was prepared with reference to the GB/T 17671–1999 test method for the strength of the cement mortar. Fluorosilicate and cement were premixed before the preparation process. To minimize any damage to the microcapsules, the mixture was stirred slowly for one minute, then molded and vibrated. The size of the cement mortar specimen is 40 mm × 40 mm × 160 mm. The content of microcapsules is 1%, 3%, 5%, and 7% of the cement content, and the proportion of fluosilicate imitating sodium silicate mortar is tentatively determined as 15% of the content of microcapsules. A blank group of cement mortar samples was also prepared for comparison.

### 2.3. Tests for Mechanical Properties and Strength Recovery

**Figure 2:**
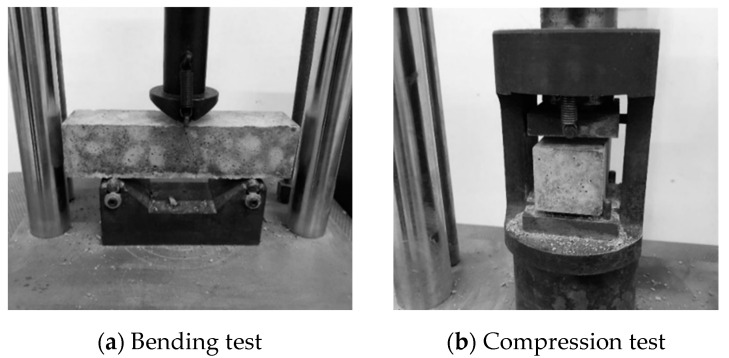
(**a**,**b**) Experimental setups for bending and compression tests.

The experimental setups used for bending and compression tests are shown in Figure 2. The age of the cement mortar is generally 7 days, 14 days, or 28 days. There is an obvious retarding effect on setting time in the microcapsules used in this test material, so the flexural compressive strength of the cement mortar was measured at 14 days and 28 days.

The residual strength of mortar specimens after complete failure can often reach 80% of the original strength, indicating that the specimens after complete failure retain a certain structural bearing capacity and the possibility of self-healing. The recovery rate of compressive strength was determined in this study in the form of secondary compression. The first batch of completely damaged specimens was placed into water for 7 days and 28 days before secondary compression. The repair rate of compressive strength = (*x* days residual compressive strength)/initial compressive strength × 100% [36], where the “healing rate of compressive strength” refers to the percentage of the healed strength of the specimen to the strength of the original specimen.

## 3. Morphology and Composition of Microcapsules

There are many characterization parameters of microcapsules, such as permeability constant, wall strength, particle size, and wall thickness of microcapsules [37,38,39]. The research object of this experiment is self-healing microcapsules in concrete, so special attention was given to the experimental performance of self-healing microcapsules at the macro scale with analysis of only the basic characteristics. Microcapsules with a particle size of about 1200 μm were selected for scanning electron microscopy (SEM, Hitachi scientific instruments (Beijing) Co., Ltd., Beijing, China) and composition analysis to further evaluate their particle size. At the same time, the thickness of the capsule wall and the element composition of the capsule core and wall were determined as basic theoretical data for further experimental research and to preliminarily verify the feasibility of adding microcapsules into concrete.

**Figure 3:**
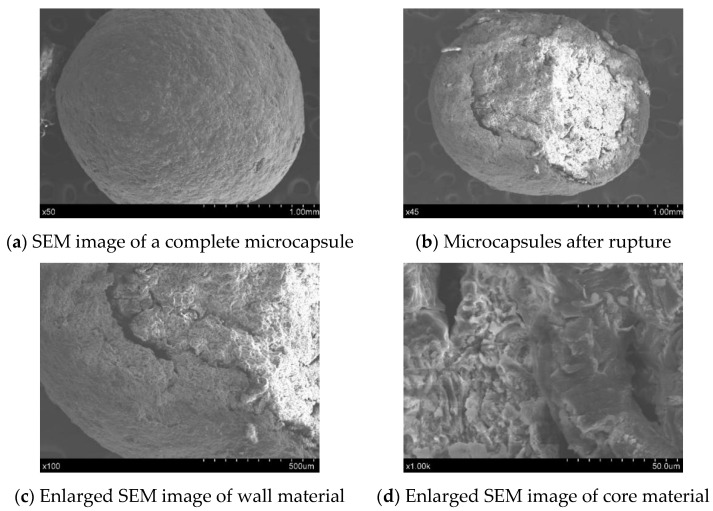

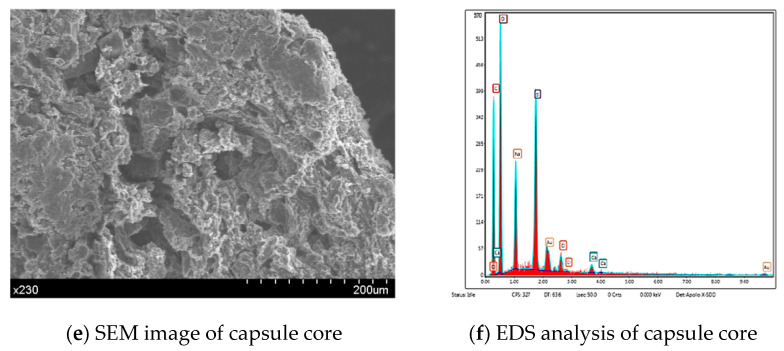
(**a**–**f**) SEM and EDS analysis of microcapsules.

A Hitachi s-3400n tungsten filament scanning electron microscope and energy dispersive spectrometer were used to study the appearance of microcapsules. As shown in Figure 3, the overall shape of the microcapsule is a regular sphere with particle sizes ranging from 1000 μm to 1200 μm. The surface is covered by coarse cellulose, and the structure is relatively compact, which can basically ensure the closure of the microcapsule in the unbroken state.

The self-healing microcapsule is mainly designed to support concrete in a hydraulic environment. After cracking, the wall material softens in the water environment so that it is more sensitive to cracks. The microcapsules prepared in this experiment satisfy the design requirements of self-healing microcapsules for concrete and show the essential self-healing mechanism.

The SEM images of the microcapsules shown in Figure 3 indicate that the material of the core and the wall of the microcapsule have obvious distinctions. The structure of the core material is not as dense as the material of the microcapsule wall. The coarse and uneven structure of the sodium silicate crystal can also be observed. The composition of microcapsules without cement suggests that the mass fraction of the carbon element is roughly consistent with the total content of cellulose in the microcapsules. The proportion of silicon and sodium is close to that of sodium silicate, and the proportion of sodium silicate is roughly the same as that of sodium silicate in the composition. Thus, sodium silicate uniformly exists in the core material.

## 4. Mechanical Properties of Microcapsule-Based Self-Healing Cementitious Composites

### 4.1. Effects of Microcapsule Content on Compressive and Flexural Strength

**Figure 4:**
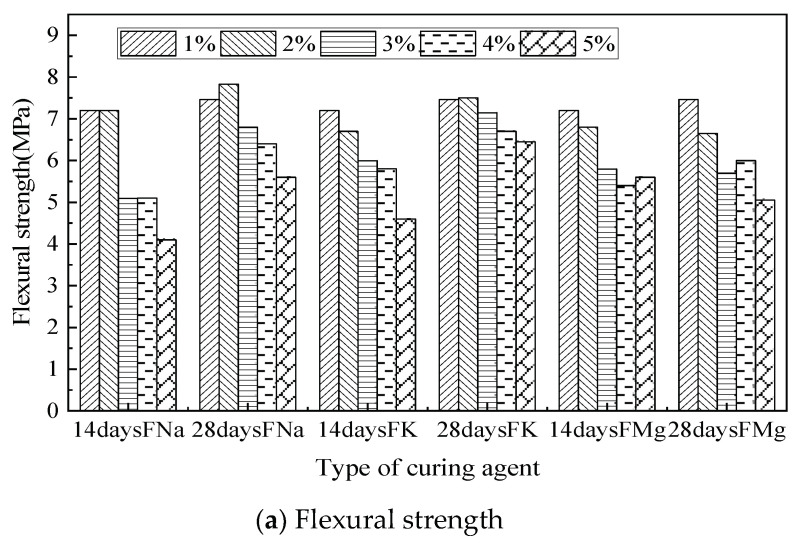

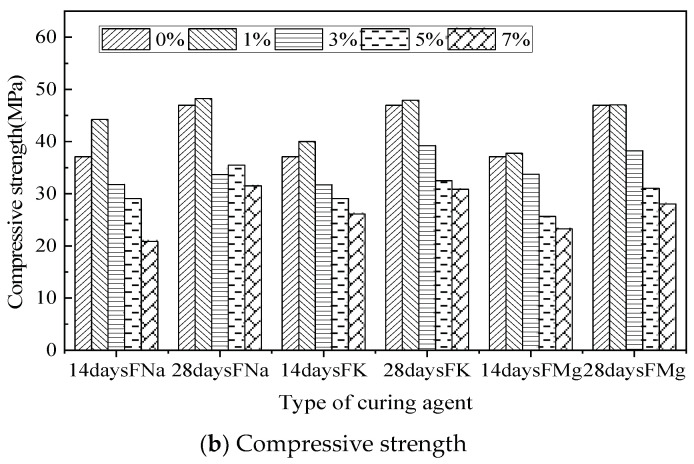
(**a**,**b**) Compressive and flexural strength of microcapsule self-healing cement specimens.

The flexural and compressive strength of self-healing concrete with FNA, FK, and FMG as curing agents in different dosages are shown in Figure 4. The flexural strength and compressive strength bar graph shows that the strength of the specimen is the lowest when the content of microcapsule dosage is 7%. As the weak phase in concrete, the microcapsule has an obvious weakening effect on the concrete when it exists in high proportions, and the specimen is more likely to be destroyed. An increase in microcapsules also decreases the proportion of cement, the main cementitious material, which exacerbates this phenomenon. The flexural compressive strength of 1% microcapsules is 2% to 5% higher than that of ordinary mortar. The addition of microcapsules also involves a small amount of fluosilicate curing agent, which has a certain strengthening effect on cement-based materials when the dosage is low [40]. The microcapsule also contains a certain amount of cellulose, which is likely to be decomposed into sugars in the process of high-temperature drying. Sugar not only retards the setting of concrete but also improves its strength [41]. Damaged microcapsules can also absorb a certain amount of water; the loss of water will reduce the water-to-cement ratio, increasing the strength of the matrix [42]. The difference between the strength of specimens aged 14 daysand 28 daysalso increased with the dosage of microcapsules, which may be attributable to the decomposition of sugar from cellulose. If only the compressive strength is used as the evaluation index, the optimal dosage of microcapsules should be between 1% and 3%; the strength of microcapsules decreases greatly when the dosage exceeds 3%.

The influence of three types of fluorosilicate on the strength of cement specimens was determined as well. The cement specimens with FNA show higher compressive and flexural strength than with the other two types. FNA appears to have better compatibility in the microencapsulated cement-based materials in this system.

### 4.2. Effects of Microcapsule Content on Secondary Compressive Strength of Composites

**Figure 5:**
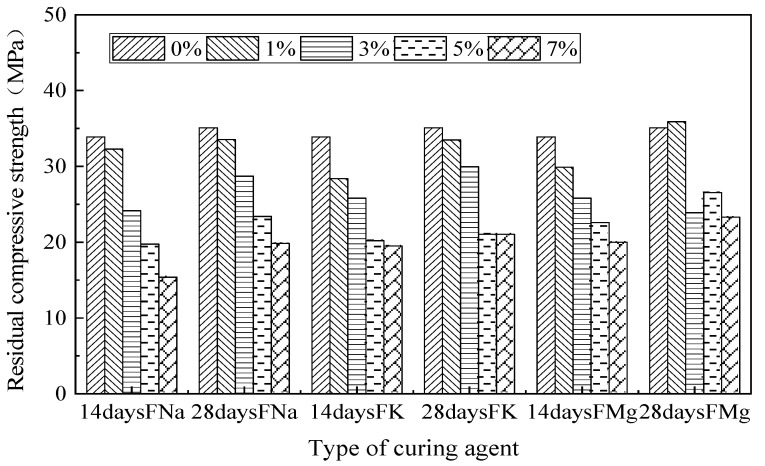
The secondary compressive strength of microcapsule self-healing cement specimen.

The secondary compressive strength of microcapsule self-healing concrete is shown in Figure 5. The change trends are similar to those shown in Figure 4. The secondary compressive strength of mortar with 1% content is lower than that of ordinary mortar, which may be related to the brittleness of cement-based materials having been increased by fluorosilicate. The specimen may retain a certain amount of secondary compressive strength at up to 70–80% of the original compressive strength. The specimens produced a large number of macro cracks, which are difficult to repair, but micro-cracks in the residual structure accompanied by macro cracks still have a certain reference value after healing.

One of the main purposes of this study is to judge whether the microcapsule self-healing concrete is effective or not. The specimens without secondary compression were soaked to healing to evaluate their self-healing performance.

## 5. Strength Healing Performance of Self-Healing Cementitious Composites

### 5.1. Before and After Healing

**Figure 6:**
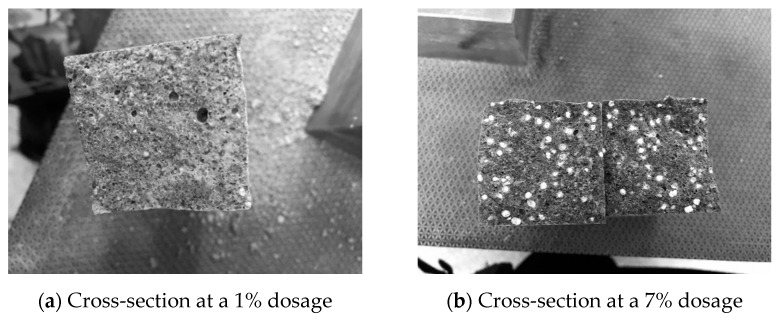
(**a**,**b**) A section of microcapsule self- healing cement specimen.

Two microcapsules with 1% and 7% content under flexural failure were selected, as shown in Figure 6. Each microcapsule was found to basically meet the requirements of the production process and was not damaged due to cement mortar production. Each microcapsule shows good bonding performance with the matrix material and can crack with the destruction of the mortar matrix, which satisfies the basic repair mechanism of self-healing microcapsule concrete.

**Figure 7:**
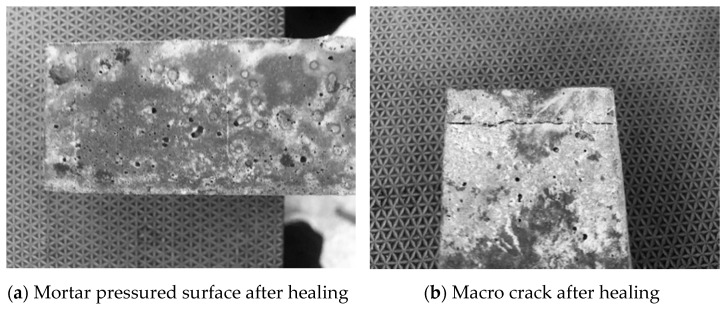
(**a**,**b**) Microcapsule self-repairing cement specimens after healing.

The healed cement mortar specimen is shown in Figure 7. A large number of white crystalline substances were produced at the self-healing mortar of microcapsules. These substances can adhere to cracks. Some of the substances may be cellulose from the rupture of microcapsules, while some are healing products generated by sodium silicate and fluorosilicate.

### 5.2. Effect of Curing Agent on Strength Healing Property of Composites

**Figure 8:**
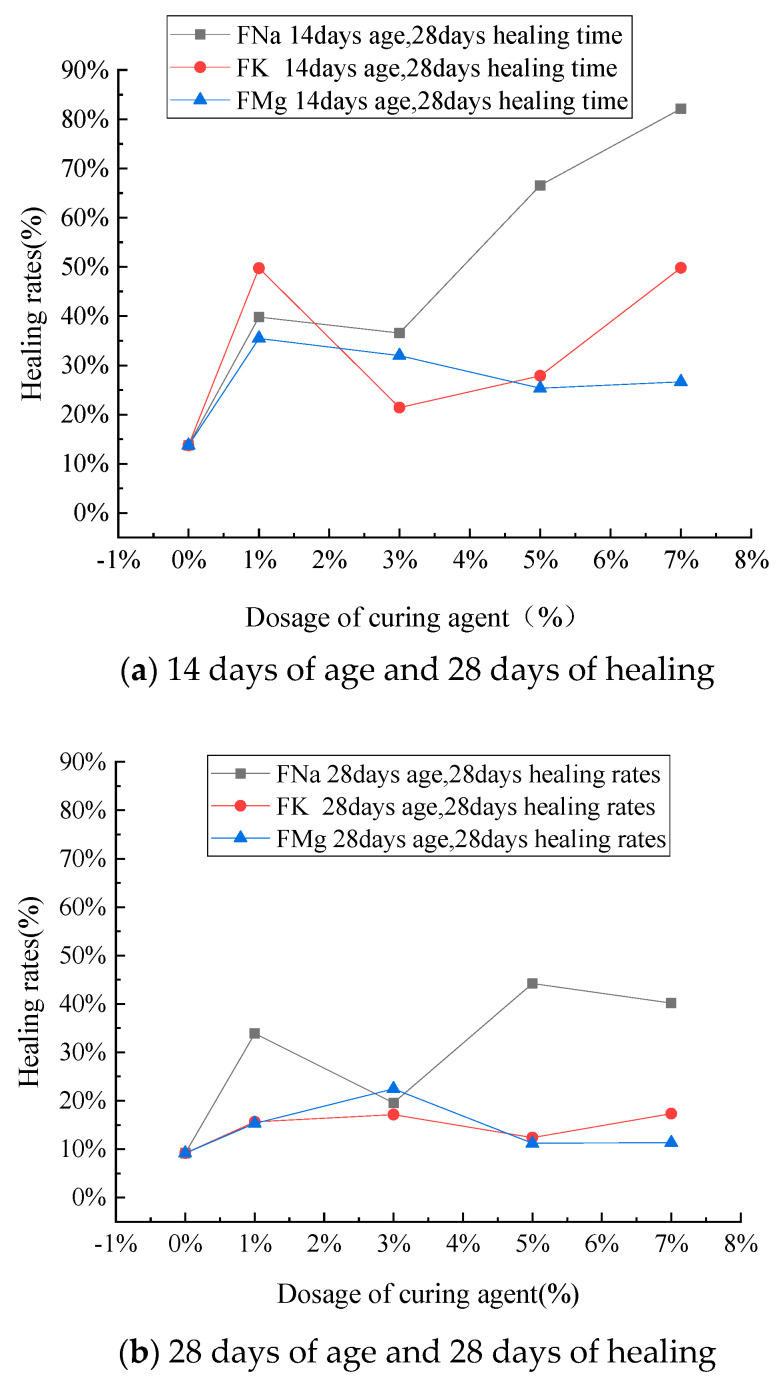
(**a**,**b**) Healing rates of specimens with three different curing agents.

The cement mortar specimens with 28 days of healing time were selected for analysis. The healing rate of compressive strength is shown in Figure 8. The healing rate of the microcapsule self-healing mortar is higher than that of ordinary mortar, which demonstrates a strong healing effect. Among them, FNA as a curing agent has a higher healing rate in most of the mortar samples with the same dosage, which indicates that FNA as a curing agent has a better healing effect than the other fluorosilicates. The healing rate of the specimens with FNA as the curing agent also appears to increase as microcapsule content increases. The curve of the other two curing agents changes less intensely with the content. The self-healing rate of mortar samples with FNA as the curing agent is 20–50% under the condition of 1–5%, which is about 40% higher than that of ordinary mortar.

### 5.3. Effects of Age and Healing Time on Strength and Healing Properties of Composites

**Figure 9:**
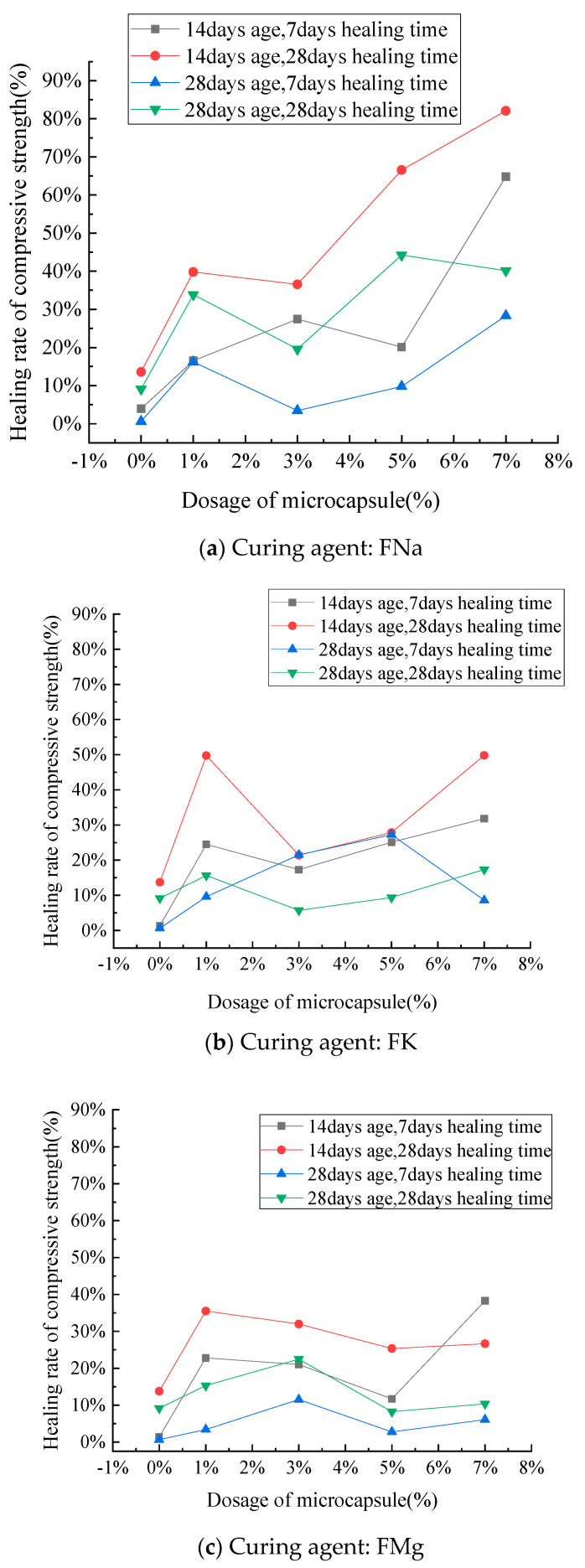
(**a**–**c**) The effects of healing time and age on healing rate.

As shown in Figure 9, the self-healing rate of cement mortar with an age of 14 days is generally higher than that of cement mortar with an age of 28 days. This is due to the fact that there is more unhydrated cement in the mortar specimens initially, and further hydration is accompanied by the maintenance of water in the repair process. This further improves the strength of the specimens over time. The healing time is also crucial to self-healing efficiency. The strength healing rate of cement mortar healed for 28 days is significantly higher than that of the specimen healed for 7 days. If the healing time could be further extended, the effect of strength recovery would likely further increase.

The curve in Figure 9b is relatively discrete, possibly because secondary compression and healing were reached by complete failure. The macro cracks produced by the mortar are more difficult to control and heal, so the broken line trend does not readily stabilize. The figure also shows that the strength recovery increases as microcapsule content increases, but there is a certain decrease in some parts that may be related to the randomness of crack occurrence and the difficulty of self-healing under complete failure. The specimen is weaker, and failure is more intense when microcapsule content is high, so the specimen aged 14 days appears to be significantly damaged. The strength of the specimen aged 28 days is higher than that of the specimen aged 28 d, and the degree of damage is relatively small, so the broken line trend is relatively stable.

## 6. Conclusions

Self-healing cement-based materials were prepared in this study using microcapsules. The reasonable dosage of microcapsules was determined, and the self-healing performance of the microcapsule-containing system was tested with various curing agents. The conclusions of this work can be summarized as follows.

(1) The microencapsulated self-healing mortar specimens prepared in the experiment showed strong healing performance. The strength healing rate obtained by secondary compression was higher than that of ordinary mortar specimens. The specimen may retain a certain amount of secondary compressive strength at up to 70–80% of the original compressive strength.
(2) The microcapsules and curing agent enhanced the flexural and compressive strength of mortar specimens at relatively low contents. The optimal microcapsule content in terms of compressive strength is 1–3%. When the microcapsule content reached 7%, the strength of the specimen decreased by about 30%.
(3) Sodium fluorosilicate is better suited to the microcapsule self-healing cement-based system than the other two fluorosilicates tested in this study. Potassium fluorosilicate and magnesium fluorosilicate as curing agents showed similarly poor healing performance. Sodium fluorosilicate is an effective curing agent that should be prioritized for further development. The self-healing rate of mortar samples with FNA as a curing agent is 20–50% under the condition of 1–5%, which is about 40% higher than that of ordinary mortar.
(4) Healing time significantly influenced the self-healing system of the microcapsule. Mortar specimens repaired for 28 days performed significantly better than those healed for 7 days.

## Figures and Tables

**Table 1:** Physical properties of cement.

Specific Surface Area (m^2^/g)	Initial Setting Time (min)	Final Setting Time (min)	Water Requirement of Normal Consistency (%)	Boiling Stability	3 days Compressive Strength (MPa)	28 days Compressive Strength (MPa)
345	140	260	26.5	qualified	27.1	42.5

**Table 2:** Chemical components of cementitious materials.

Components	SiO_2_	Al_2_O_3_	Fe_x_O_y_	CaO	MgO	SO_3_	K_2_O	Na_2_O	LOI
Content	21.6%	4.3%	2.6%	65.8%	1.2%	1.6%	0.7%	0.4%	1.8%

## Data Availability

No new data were created or analyzed in this study. Data sharing is not applicable to this article.
